# Trichloracetic Acid a Specific in Vincent’s Angina

**Published:** 1919

**Authors:** 


***Trichloracetic Acid a Specific in Vincent's Angina.*** By Dr. T. J. Gallaher. *The Laryngoscope*, July, 1918.

The author brings the following remedy for Vincent's angina to the notice of medical men.

Trichloracetic acid should be applied pure by means of a small applicator wound with cotton and dipped into the liquid. The excess is removed by touching it against an ordinary blotter, and the acid is carefully applied to the entire area affected. In case of the tonsils, the acid should be carried on a thin applicator to the depth of each crypt involved, in addition to the surface application. After the parts are turned white it should be neutralized in one or two minutes by the application of a saturated solution of sodium bicarbonate. The application of the acid and the neutralization may be repeated in 2 or 3 days if necessary. The results will be dependent upon the thoroughness of the application. Two applications have been rarely required. An excess of acid must not be used, and none must be permitted to fall into the larynx.

